# A Depth-Based Hybrid Approach for Safe Flight Corridor Generation in Memoryless Planning

**DOI:** 10.3390/s23167206

**Published:** 2023-08-16

**Authors:** Thai Binh Nguyen, Manzur Murshed, Tanveer Choudhury, Kathleen Keogh, Gayan Kahandawa Appuhamillage, Linh Nguyen

**Affiliations:** 1 Institute of Innovation, Science and Sustainability, Federation University Australia, Churchill, VIC 3842, Australia; thaibinhn@students.federation.edu.au (T.B.N.); t.choudhury@federation.edu.au (T.C.); k.keogh@federation.edu.au (K.K.); g.appuhamillage@federation.edu.au (G.K.A.); 2 School of Information Technology, Deakin University, Burwood, VIC 3125, Australia; m.murshed@deakin.edu.au

**Keywords:** depth sensing, memoryless planning, safe flight corridor, drones, UAV

## Abstract

This paper presents a depth-based hybrid method to generate safe flight corridors for a memoryless local navigation planner. It is first proposed to use raw depth images as inputs in the learning-based object-detection engine with no requirement for map fusion. We then employ an object-detection network to directly predict the base of polyhedral safe corridors in a new raw depth image. Furthermore, we apply a verification procedure to eliminate any false predictions so that the resulting collision-free corridors are guaranteed. More importantly, the proposed mechanism helps produce separate safe corridors with minimal overlap that are suitable to be used as space boundaries for path planning. The average intersection of union (IoU) of corridors obtained by the proposed algorithm is less than 2%. To evaluate the effectiveness of our method, we incorporated it into a memoryless planner with a straight-line path-planning algorithm. We then tested the entire system in both synthetic and real-world obstacle-dense environments. The obtained results with very high success rates demonstrate that the proposed approach is highly capable of producing safe corridors for memoryless local planning.

## 1. Introduction

In recent years, while significant advancements in aerial robotics have been opening up various applications across industries, including inspection, search and rescue, surveillance, exploration, and more [1,2,3,4], autonomous navigation in complex environments still presents challenges, especially in cluttered and obstacle-dense environments. The localization and mapping of aerial robots can be subject to state estimation error and sensor fusion drift [5,6,7]. On the other hand, maintaining a high-resolution map in real time demands high computational resources [8]. To avoid those limitations, existing research has explored computationally efficient approaches that utilize only the most recent sensor data and do not maintain any global map, namely mapless [9,10,11] or memoryless planning [12,13]. Memoryless planning is similar to classical sensor-based planning [14,15], which does not have complete knowledge of the environment a priori. The robot relies on its integrated sensors for local perception and planning. These approaches are inspired by human behavior, which cannot remember environmental features over a long time but can perceive the local risks or uncertainties and quickly react to them.

However, due to its inherent short observation horizon, the overall planning performance is degraded, and the computational efficiency may not be at its best. Specifically, as a local planning algorithm, memoryless planners do not guarantee planning completeness since they can be trapped in a dead end [13]. Learning-based methods [16,17,18], a typical type of memoryless algorithms, suffer from prediction uncertainty, which can lead to collisions with obstacles, especially end-to-end learning [19]. On the other hand, classical local planners usually utilize sampling techniques in the local occupancy grid [20,21,22,23,24] or in sensor raw data [12], consuming considerable computing resources due to local occupancy fusion or a sampling job.

We aim to design a method to combine the advantages of both approaches and overcome their weaknesses. This work focuses on delivering a safe flight corridor (SFC) generator, utilizing a learning-based object-detection engine. We exploit robustness from a learning model to generate explicitly separated polyhedral corridors, which are beneficial for path planning. We then adopt a depth-based verification procedure to discard SFC that would result in a collision. Although this step can be over-conservative, it eliminates false predictions from a learning object-detection model, ensuring our generated SFCs are collision-free.

Our contributions can be summarized as follows:

(1) We propose a hybrid safe flight corridor generation technique that utilizes only depth images. It produces less-overlapping corridors compared to a depth-based inflating method.
(2) We propose a memoryless planner employing the above-generated corridors to navigate a quadrotor autonomously through an obstacle-dense environment to a destination without employing any map or external global planners. The entire planning system can run in real time with fully onboard sensing and computation.
(3) We implemented and validated the proposed method on an actual fully autonomous quadrotor system. Real-world flight tests show that our methods can effectively navigate a quadrotor through cluttered scenarios.

## 2. Related Works

### 2.1. Corridor-Based Trajectory Planning

In recent years, corridor-based trajectory planning techniques have gained popularity as a means of ensuring safe quadrotor flights. Liu et al. [23] rely on an underlying global occupancy grid that can be constructed from sensor data such as laser range finder, stereo cameras, or RGB-D sensors. A graph search algorithm can then find a valid collision-free path in the grid. Utilizing OctoMap [25], an occupancy map, Chen et al. [22] use free cubes in the map as the spatial constraints. Ren et al. [26] first build a KD-Tree with the obstacle point cloud, then inflate sphere-shaped corridors by a sampling process. These works must fuse an underlying occupancy grid upfront before generating SFCs.

Utilizing only the latest depth image, Bucki et al. [12] invented an algorithm for inflating rectangular pyramid corridors when needed to check for collision in a sampled polynomial trajectory. Since they uniformly sample in the surrounding space and try to inflate a new pyramid around that sampled point, the generated SFC collection can overlap much and become unnecessary constraints of the same spatial boundary for path planning. Moreover, the random sampling technique itself is also not quite computationally efficient.

### 2.2. Learning-Based Local Planning Limitation

The end-to-end method [27] takes a simple but promising navigation pipeline. Environmental information measured by a set of sensors comes directly to a black-box model that can output navigation commands to the robot. In several architectures, outputs are valid control signals to the robot’s actuators [19,28]. Since an end-to-end model is a learning-based network, this type of architecture suffers from its uncertainty.

Another learning-based method is probabilistic trajectory prediction [16,17,18], which was based on underlying collision probability to choose the likeliest collision-free trajectory. Hence, even when prediction accuracy is very high, the robot may still collide or come to a dead end, especially navigating in an obstacle-dense environment, shallowing the success rate. The authors of [17,18] produced significant hand-engineered data for training their model. Expert training data can greatly affect the prediction model’s performance. Nguyen et al. [16] exploit a flight simulator and an autonomous collecting mechanism to acquire their data point, which is a series of sequential synthetic depth images accompanied by the robot’s action and corresponding collision status for each image. Since a data point encrypts the dynamic state information of the robot, it may suffer from synchronization errors and state estimation uncertainty. On the other hand, since their dataset design relies on collision data points (having the robot collide), they are not likely to produce actual data but simulation data only for model training.

Furthermore, their system needs an external global planner’s instruction to operate and can become trapped when no action sequence qualifies the probability threshold (all actions will lead to a collision).

### 2.3. Free Space Segmentation

Image segmentation is an essential and widely researched topic in computer vision and image processing, and it can provide the necessary information to separate free space [29]. Image segmentation aims to partition the image into meaningful regions, preserving the boundaries and properties of the objects in the scene. Free space segmentation is one of its applications, referring to the process of using a computer vision model to divide images into traversable space and occupied space (obstacles) [29,30,31,32]. The model’s input can be monocular or stereo images; the output is pixel-wise segmentation.

One of the limitations of free space segmentation is that it cannot produce geometrical representations of the free space, which are often required by collision-checking algorithms or trajectory optimization techniques in autonomous navigation. Free space segmentation algorithms typically produce a binary or pixel-wise map separating free space from the occupied space. However, this information alone may not be sufficient for local planning as it is challenging to mathematically model the safety constraints from non-geometrical forms. Another limitation is the possibility of false segmentation, particularly in challenging environments such as urban scenes and natural landscapes. The algorithm’s output needs to be verified to ensure that it is accurate and reliable.

## 3. Problem Formulation

**Figure 1:**
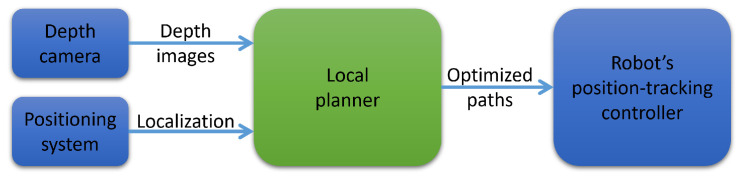
Local planner architecture.

The broader problem in this paper will consider a quadrotor traveling in an obstacle-dense space from a start to a destination without access to the map of the environment. The only sensing information is real-time depth data from a stereo camera constrained by its range and field of view (FoV). We assume that the robot is provided with the real-time position of itself, the starting point, and the destination. The localization data can be obtained by either integrated Global Navigation Satellite System (GNSS) modules or other local positioning systems. We aim to design a learning-based memoryless planner capable of guiding the robot through obstacles to reach the goal. Let $O_{t}$ and $G_{t}$ are the world-frame location of the robot and the destination at the current time *t*, $m_{t}$ is the current depth image, and the solution in the form of a straight-line path $\vec{O_{t}P_{t}}$ while $P_{t}$ is the current path’s endpoint. Since our memoryless planner’s solution only depends on the current depth image $m_{t}$, we drop the subscript *t* where convenient to simplify the notation. Then, the formal problem will be finding an optimized collision-free path $\vec{OP}$ for each of every planning sequence that allows the robot to travel safely toward the destination, given (O,G,m). We propose the local planner architecture as illustrated in Figure 1. The robot’s real-time location O is provided by a GNSS module. The goal location G is prefixed and stored as a constant in the local planner. The generated optimized path will be used as a position reference for the robot’s position-tracking controller.

## 4. Free Space Detection

For classical approaches, the difficulty of the SFC generation problem increases with the environment’s clutteredness. We want to generalize the free space partitioning problem for any arbitrary scenarios. Instead of extracting safe corridors by sampling [26] and geometrical inflating [12] as classical approaches, we employ a learning-based network for detecting free space directly from depth images, which requires an equal amount of computing resources for all scenarios.

### 4.1. A Hybrid Approach

**Figure 2:**
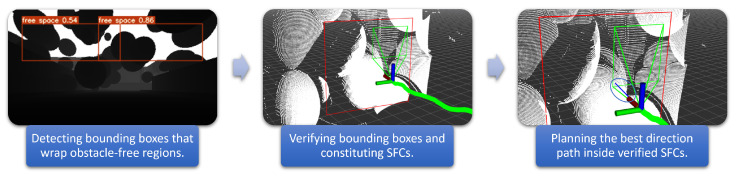
Free space detection planning flow.

We propose a hybrid approach to retain the advantages of a learning-based method and remove its false predictions by a lightweight depth-based verification. First, we train a deep-learning model to predict bounding boxes that wrap obstacle-free regions directly in a depth image. Next, we verify the predictions by rejecting collided bounding boxes at every depth and then searching for the maximum depth possible for the remaining. Finally, we plan the best direction path (blue segment) toward the goal that satisfies SFC constraints. Therefore, no sampling job is needed. We call the process a hybrid because the verification is a non-learning technique, involving a classical search-based algorithm. The workflow of the planner is outlined in Figure 2.

### 4.2. Safe Flight Corridor Constitution

**Figure 3:**
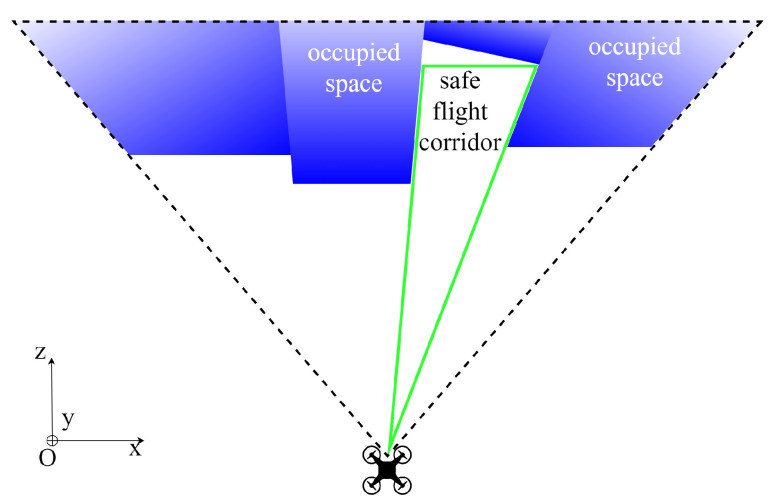
Visualization for the top view of the depth point cloud inside the FoV.

We define our SFCs in a pyramid-shaped form to take advantage of the depth image characteristics. One of the features of depth images is that, when deprojecting it into space, a segment connecting the optical center to any pixel does not intersect any segment connecting the optical center to any other pixel. Therefore, any plane parallel to the sensor frame and not obscured by any pixels will be collision-free. Additionally, any segment connecting the optical center (the robot’s position) to any point on that plane will also be collision-free. If the plane is rectangular, we can constitute a rectangular pyramid SFC with its apex located at the robot’s location O, and the base perpendicular to the z-axis of the depth camera-fixed frame, as visualized in Figure 3.

**Figure 4:**
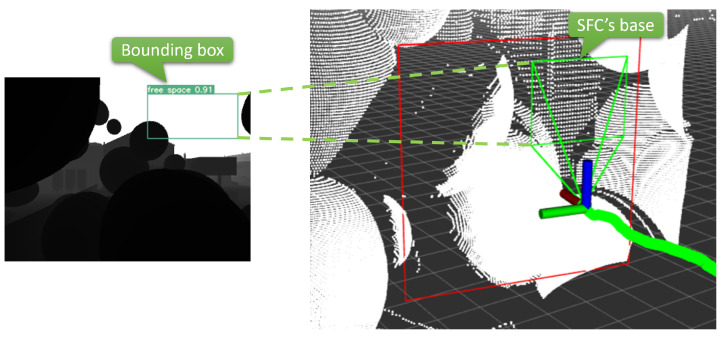
An SFC constitution from a predicted bounding box.

To archive the mentioned pyramid SFCs, we need to generate its rectangular base in the space. Since we predict SFC only in the current depth image, the pyramid base will also be in the camera’s FoV and can be specified by its projection in the image frame. Let $bb=(x_{1},y_{1},x_{2},y_{2})$ denotes the bounding box that represents the SFC base’s projection where in this case, $x_{1}$, $y_{1}$, $x_{2}$, and $y_{2}$ are the pixel coordinates of its left, top, right, and bottom in the image frame. Our object detector aims to predict bb so that the corresponding constituted SFCs are collision-free. The flow of the SFC constitution from a predicted bounding box in the image frame is visualized in Figure 4.

It is noted that the network learns to perceive safe corridors purely from the depth values of neighborhood pixels. Therefore, the quality of generated SFCs and the underlying planning algorithm does not depend on the features or materials of the environment if depth estimation is accurate enough. In other words, foliage in a rainforest or mirrored objects will be perceived as the form of depth frames and will be treated equally when they are fed to the model. That also means the network can label a 2D bounding box containing faraway obstacles as free space. This makes sense because we can still extract sufficient traversable SFCs between the robot and unreachable obstacles. This generalization turns out to be effective as the trained model can generate a safe corridor from both obstacle-dense and sparse situations with the same amount of computation.

On the other hand, the training also benefits from that formulation as it requires only one depth image and corresponding SFC annotations to constitute a data point. Specifically, the training data only needs to tell how deep and big the SFCs are via a depth image and corresponding annotations. The trained model will take one depth image as an input and output bounding boxes bb representing projections of SFCs.

This work assumes that the accuracy of the depth estimation is perfect, and the discussion on it is beyond this project’s scope. In reality, the depth estimation accuracy of popular commercial cameras is reliable enough for this application. For example, an Intel^®^ RealSense™Depth Camera D435 has an accuracy of under 2% of depth at 2 m (Intel^®^ RealSense™Depth Camera D435 Tech Specs https://www.intelrealsense.com/depth-camera-d435/, accessed on 1 June 2023) in the z-axis measured as out of the factory. This amount of uncertainty in depth estimation is acceptable and can be easily eliminated by adding safety margins on training data.

### 4.3. Supervised Learning and Conservativeness

It is necessary to provide a deep-learning object detector with adequate training data that fully capture our object’s features [33]. For a standard 2D annotation describing the ground truth of an object, the features will be the size of the bounding box and the pattern inside, which will be translated as pure depth values of pixels in our problem. Those features will be determined by specifying how far the bounding boxes, which are projections of ground-truth SFCs in our case, should stay from the closest obstacles and how deep they will be to remain collision-free. Thus, labeling SFCs using these criteria is equal to annotating patterned objects.

Supervised learning systems work best with fully labeled data points, meaning free space (in the form of an SFC) should be exhaustively extracted from the depth image. However, many SFCs that satisfy the abovementioned criteria would be in an arbitrary depth image of cluttered scenarios, resulting in many bounding boxes overlapping each other. This scenario of annotation is not an ideal training data point for a deep-learning object detector since it confuses generalization [34]. Thus, we label only one largest SFC per depth image. Although this approach is conservative since certain parts of the free space stay unlabeled (weak labeling [35]) in a very cluttered environment, it benefits the generalization of the deep-learning model. We will demonstrate that the result of training this dataset with supervised learning is practical enough for applications. In this paper, the deep-learning model Yolov7 [36] has been selected as the object-detection engine due to its status as the best object-detection model at the time of writing.

### 4.4. Data Collection and Training

**Figure 5:**
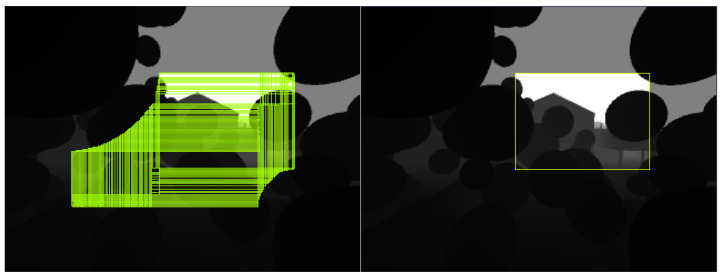
Depth-based inflating methods produce highly overlapped SFCs (**left**) unsuitable for training data points. We label only one largest bounding box (**right**) for training the free space detection model.

We collect training data from both the flight simulator and the actual environment. We run the planner in our previous work [37] in the Flightmare drone simulator [38] and capture depth images at the resolution of 320 × 240 from a simulated onboard camera. We then label a bounding box representing the base of a rectangular pyramid SFC by a modified version of the depth-based inflating algorithm in [12] in which we brute-forced search all pixels in the frame. That exhaustive inflating method generally outputs overlapping bounding boxes in the frame without concerning obstacle density, as illustrated in Figure 5.

This annotation type cannot be used for training because it does not imply an explicit feature for the SFC object. Labeled SFCs in an image should not be overlapping and represent separated free space at a constant and specific depth. Therefore, we label only one bounding box in an image. We modify the depth-based inflating algorithm to exhaustively search all the pixels at a depth of 1 m, inflate all the possible rectangular pyramids, and then peak the one with the largest base. The largest base will be extracted as a bounding box representing an SFC.

**Figure 6:**
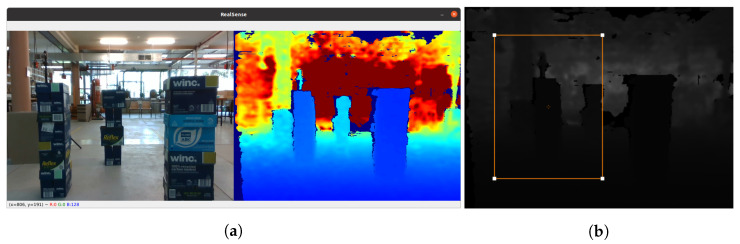
Actual depth images collected from the indoor environments will be labeled. (**a**) Actual RGB and corresponding heatmap-based depth frame. (**b**) Labeled depth frame.

We also collect actual depth images with a RealSense D435, a depth camera from Intel. The camera has an FoV of 87${}^{\circ }$× 58${}^{\circ }$ for depth sensing. The maximum frame rate at the resolution 320 × 240 is 90 frames per second (fps). Actual depth frames are also captured at the resolution of 320 × 240. A person holding the camera walks around the indoor and outdoor environments to obtain sample depth frames, including clear spaces and obstacle-filled scenarios, as shown in Figure 6.

For each synthetic and real image collection, we produce 36,000 frames, split into a train, validation, and test set with a ratio of 90:8:2.

Applying an autonomous labeling mechanism, we can save a huge resource in producing a hand-engineered data process used in [17,18]. Our method does not need to encrypt any robot’s dynamic information into training data. Therefore, we can collect data from various scenarios and configurations, simulated and real frames, without worrying about synchronization, estimation inaccuracy, or vehicle damages. Furthermore, our free space detection approach accepts data points containing only one image and its annotations, much lighter than other sequence data points in collision probability prediction approaches.

**Figure 7:**
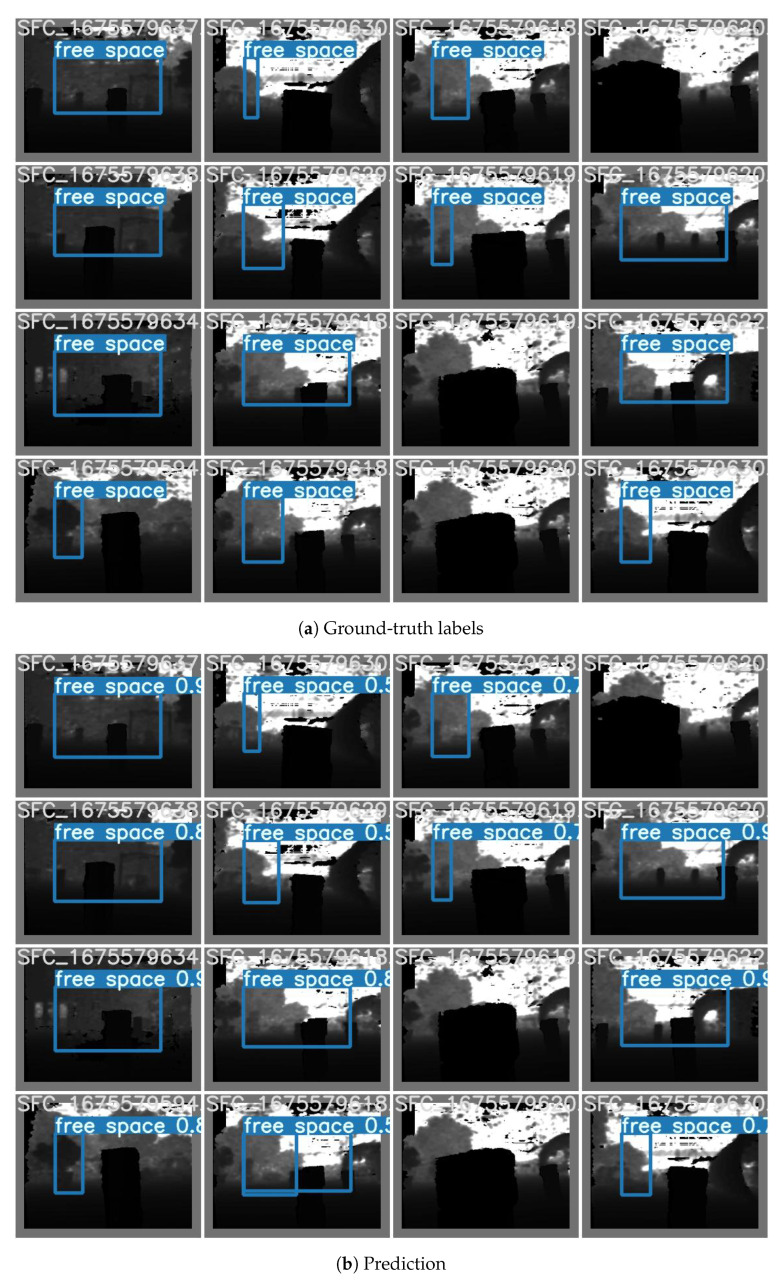
Prediction results of the trained model in the test set.

Training hardware must support the Pytorch framework (Pytorch https://pytorch.org/, accessed on 1 June 2023) as required by Yolov7. We train the model in our available local server with an NVIDIA GeForce GTX 1080 Ti 11 GB GDDR5X. Our model will be transfer-learning trained from the yolov7-tiny.pt, which is the checkpoint of a Yolov7 tiny pre-trained model with the COCO dataset. The training configuration will be 64 samples per batch and 150 epochs. Given the Intersection over Union (IoU) threshold of 0.5, the trained model achieves a prediction accuracy of 94.9%, recall of 92.9%, mean average precision at IoU 0.5 (mAP@0.5) of 96.1% and mAP@0.5:0.95 of 80.9% on the test dataset. Free space prediction results on the test set are visualized in Figure 7. Predicted SFCs are reasonably similar to the ground truth.

**Figure 8:**
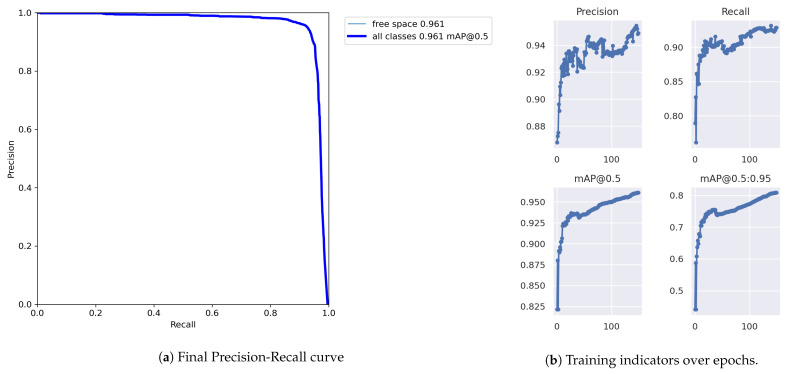
Training results.

As illustrated in Figure 8, the precision, recall, and mAPs converged to stable values over training epochs. Since we have only one class of object, the precision-recall (P-R) curve for the free space class coincides with the global P-R curve for all classes. The P-R curve profile shows that the precision approached 1 when the recall approached 0 and vice versa, maximizing the integral of the area under the P-R curve, which is the mAP@0.5 of 96.1%. It takes about 40 ms to infer an image on Jetson Xavier NX and an average of 8 ms on a laptop GPU.

### 4.5. Free Space Prediction and Verification

**Figure 9:**
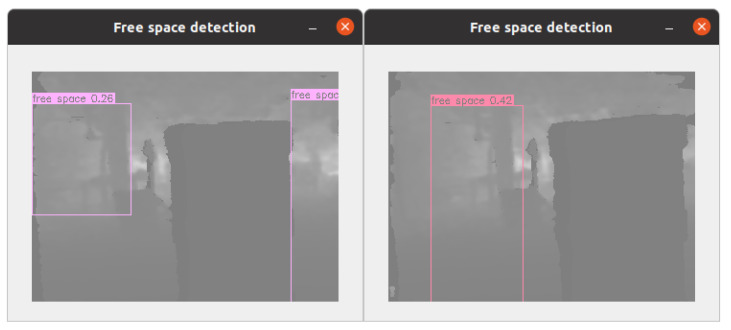
Illustration of several predictions with corresponding scenes.

Follow the flow described in Figure 2, the trained model will predict the bounding box in the depth matrix as illustrated in Figure 9. We search for the closest pixel’s depth value $Z_{min}$ to the robot in the bounding box and then decrease it by the quadrotor radius *R* to receive the max depth value of the SFC’s base as follows,

(1) $$D_{max}=Z_{min}-R$$

Our SFC will be a rectangular pyramid formed by the detected bounding box at a previously calculated depth value $D_{max}$ as the base, and the apex is in the robot’s location. We will discard a constructed SFC if its base is smaller than the quadrotor size. We can verify that by deprojecting the bounding box to the space to obtain the actual size of SFC’s base, using the corresponding depth value and the camera’s intrinsic matrix. That verification process ensures that our generated SFCs are collision-free and spacious enough for later path planning.

## 5. Local Planning Algorithm

### 5.1. Local Planner

Within a collective of SFC constraints, finding the optimal path with respect to an arbitrary objective function may not be trivial. However, by formulating a particular optimization problem, we can end up solving a trivial one. Specifically, a direction reward function will be proposed and discussed later. We employ a 25 Hz straight-line path planner that produces an optimized reference path for each planning cycle. As stated in the problem formulation, each path $\vec{OP}$ is specified by the segment connecting the current robot location O and an endpoint P in the space. The goal is to find the endpoint P so that $\vec{OP}$ is collision-free and has the best direction reward to the goal. A position-tracking linear feedback controller will execute generated paths in a receding horizon fashion. It continuously tracks the latest reference path.

### 5.2. Best Direction Path

We apply a direction function J(P), inspired by our previous work [37], for evaluating path’s reward as follows:

(2) $$\begin{array}{cc} \vec{d} & =\frac{\vec{\mathrm{OG}}}{∥\vec{\mathrm{OG}}∥}\\ J(P) & =\frac{\vec{d}.\vec{\mathrm{OP}}}{∥\vec{\mathrm{OP}}∥} \end{array}$$

(3) $$\begin{array}{cc} \underset{P}{arg max}\; & J(P) \end{array}$$

where O, G, and P are the world-frame positions of the vehicle, the goal, and the best path’s endpoint we need to find, respectively. $\vec{d}$ is the vector of exploration direction. This vector-based function corrects the velocity vector to the desired direction. In our proposed system, $\vec{d}$ will be the unit vector from the vehicle’s current position to the goal. The reward is simply the dot product of the exploration vector $\vec{d}$ and the unit vector of the path, i.e., the direction of a better path will align closer to the goal direction. With *p* and *g*, respectively, as the projections of P and G in the image plane, we can see that J(P) is at its maximum when the distance between *p* and *g* is minimal. With rectangular bounding boxes as constraints, our problem will be a 2D geometry issue: find the point *p* inside bounding boxes bb so that the distance |pg| is minimal. We have the coordinates of the goal and its projection in the camera frame calculated as follows,

(4) $$\begin{array}{cc} G & =(x_{G},y_{G},z_{G})\\ g & =(x_{G}*f_{x}/z_{G}+c_{x},\;y_{G}*f_{y}/z_{G}+c_{y})\\ bb & =(x_{1},y_{1},x_{2},y_{2}) \end{array}$$

where $f_{x}$, $f_{y}$, $c_{x}$, and $c_{y}$ are the focal lengths and principle point’s coordinates in the corresponding axis, respectively; $x_{1}$, $y_{1}$, $x_{2}$, and $y_{2}$ are the bounding box’s left, top, right, and bottom coordinates, all calculated in pixel unit. P and G coordinates are in meters. Our problem becomes:

(5) $$\begin{array}{cc} \underset{p}{arg min}\; & \left|pg\right|\\ s.t.\; & p\in bb \end{array}$$

**Figure 10:**
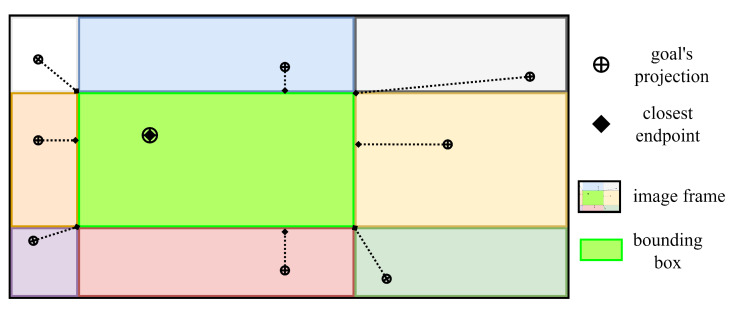
Illustration of the solution for finding *p* inside the bounding box with respect to *g*.

The solution can be found trivially by a 2D geometry approach. We divide the region inside a bounding box and the image plane by nine areas, as illustrated in Figure 10. *g* will fall into one of nine areas. *p* can be specified in three situations:

(a) If *g* falls into one of four corner areas, *p* is the nearest corner.
(b) If *g* falls into one of the four edge areas, *p* will lie on the nearest edge, specified by a perpendicular line drawn from *g* to the edge.
(c) If *g* falls into the bounding box, the solution is *g* itself.

Finally, we deproject *p* back to *P* in the space by equation:

(6) $$\begin{array}{cc} P=( & \left(x_{p}-c_{x}\right)*D_{max}/f_{x},\\ \; & \left(y_{p}-c_{y}\right)*D_{max}/f_{y},\\ \; & D_{max}) \end{array}$$

Furthermore, we apply the depth-based steering policy as in our previous work [37] to generate steering commands for several local minima motions, navigating the robot over large convex obstacles.

## 6. Implementations and Results

### 6.1. Simulation

**Figure 11:**
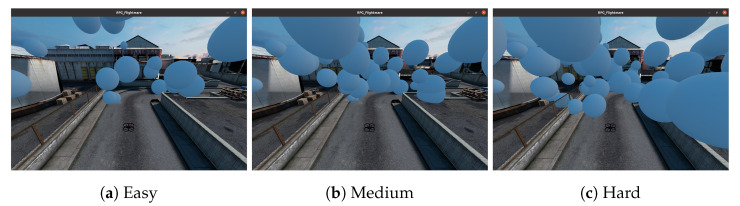
Scenarios in the auto evaluation.

We test our proposed navigation planning system, which we now call **FSD planner**—**F**ree **S**pace **D**etection planner for brevity, on a simulation system (Our simulation system source code is available online at https://github.com/thethaibinh/agile_flight/tree/fsd, accessed on 1 June 2023) from DodgeDrone Challenge 2022 [38]. The challenge is to navigate a quadrotor through the obstacle-dense area to a 65 m far ahead goal. For the convenience of auto evaluation, we modify the scenario to be a quadrotor flying through a 15 m route to a goal with obstacles along the route. The flight area is a 3D bounding box with sizes of [0, 15, −5, 5, 0, 10] (meters) in a frame system with the axis Ox and Oy parallel to the ground and Oz pointing upwards, in which the robot is at the origin, and the goal is at [17, 0, 5]. Obstacles are solid spherical balls of different diameters ranging from 0.1 to 4.0 m, stationary, and randomly generated inside the bounding box. There are three levels of challenge based on the density of balls in space: Easy, Medium, and Hard, with 29, 51, and 67 balls in the test box, respectively. Figure 11 capture a typical scenario with additional visualization of point cloud ground truth. All obstacles of the easy scenario are already included in the medium scenario, and the hard scenario has all the medium obstacles in its configuration. A trial will be labeled successful when the robot reaches the goal’s position before the timeout and does not collide with any obstacle.

**Figure 12:**
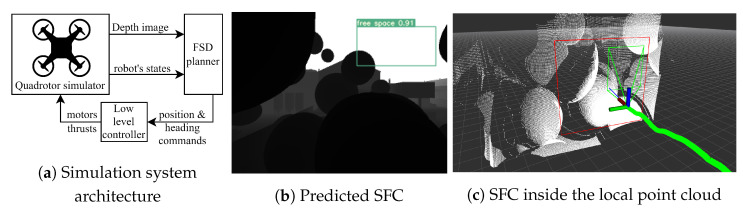
Simulation system overview and visualisation.

We train a separate model for use in the simulation by synthetic data also saved from the same simulator. We flew a couple of trials by the planner in our previous work [37] and captured synthetic depth images along the routes. We then labeled them and trained the model using the method mentioned in Section 4.4. The classical nested feedback loops [39] will be integrated as a low-level controller who receives position guidance commands in a receding horizon manner. We limit the maximum desired velocity to 1 m/s, mitigating control uncertainty because we only focus on SFC evaluation. Figure 12a describes an overview of the simulation system, mapping out the planner architecture sketched in Figure 1. All member components in the simulation system are software and will be run on the desktop environment. Figure 12b,c visualizes predicted SFCs in the current depth frame and corresponding local point cloud.

We will run the simulation flight 1000 times for each scenario on an Intel^®^ Core™i7-11800H laptop equipped with an NVIDIA RTX3070 8 GB GPU.

### 6.2. Flight Tests

**Figure 13:**
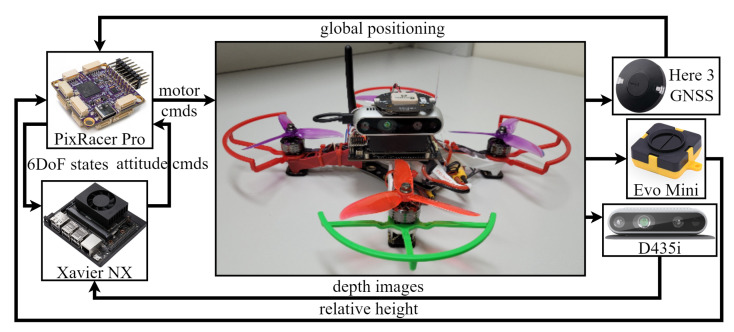
Experimental quadrotor with onboard computation and sensors.

For real flight tests, we build a 250 mm wheelbase experimental quadrotor that houses a GNSS module for obtaining global localization, a TeraRanger Evo Mini altimeter for relative altitude feedback, and a stereo camera. Mapping with the planner architecture described in Figure 1, the positioning system will be a Here 3 GNSS module, the depth camera will be an Intel RealSense D435i, and the position-tracking controller will be implemented in a companion computer Jetson Xavier NX. The quadrotor also has a PixRacer Pro flight controller for running low-level orientation tracking controllers and 6DoF state estimation. The embedded onboard computer Jetson Xavier NX runs the FSD planner and the position-tracking controller, similar to the simulation system. The drone architecture is outlined in Figure 13.

We also trained a separate model for use in the real flight test, using indoor and outdoor captured depth images. Our artificial obstacles are several columns formed by paper boxes. The obtained results show that the proposed planner can still safely guide the vehicle through scenarios of those columns.

### 6.3. Results

Polyhedral SFCs, like the pyramids we produce, will enforce multiple Euclidean planes as boundaries for our straight-line paths. Therefore, regarding computing workload, a small number of SFCs covering large areas of free space would be computationally cheaper for path planning than a collection of overlapping ones. We compare the overall intersection over union (IoU) of generated SFCs from our method with that of SFCs from the exhaustive inflating algorithm. We run two methods on a library of 600 actual depth images captured from Intel RealSense D435i. The lower the overall IoU is, the better the collection of SFCs is. Also, the higher the average max depth and volume of SFCs, the better. We can notice that the average of the deepest SFC’s depth and the average of the largest SFC’s volume from the proposed method are slightly lower than the inflating method due to the conservative verification. Although SFCs in inflating collection overlap by an IoU of 42.27%, our detected SFCs’ IoU is only 1.35% on average. The straight-line path planner runs faster on given less-overlapping SFCs generated by the proposed method than exhaustive inflating ones and produces the same average path direction reward. Comparisons are summarized in Table 1.

Given safe corridors generated by our proposed method, the path planner always finds a feasible path for the quadrotor with success rates for three scenarios are 99.7%, 99.9%, and 99.6%, respectively. All the trials were successful except for a few failed corner cases due to failures of tracking controllers. Simulation flight results are summarized in Table 2.

**Figure 14:**
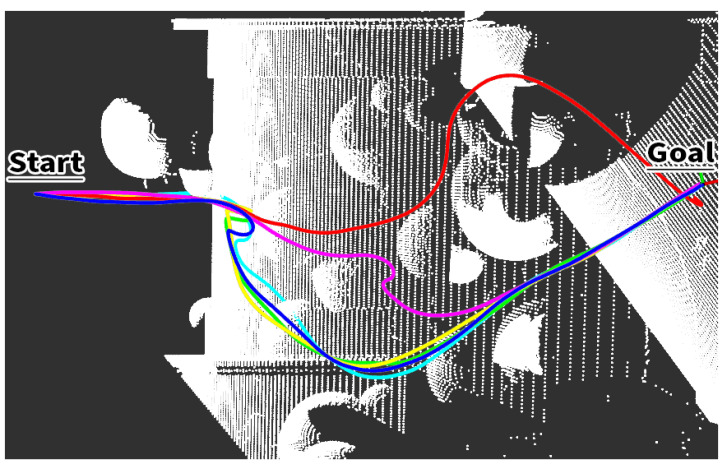
Executed trajectories from start to the goal by the robot in a medium scenario.

Figure 14 illustrates several traveled paths in the simulation Medium scenario. It can be noticed that the FSD planner can effectively generate a collision-free and smooth path for the drone to move toward the goal.

**Figure 15:**
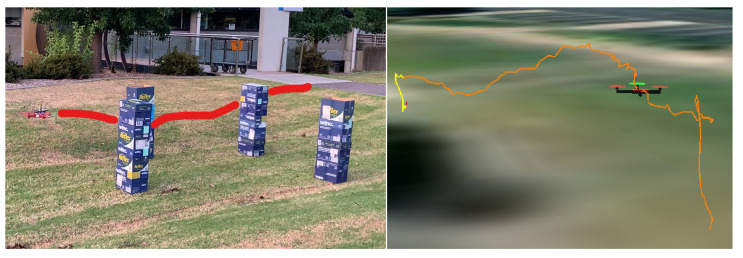
Flight test with paper boxes as obstacles.

Executed trajectory in real flight experiment has been captured in the flight log and illustrated in Figure 15. A demonstration video of how the quadrotor navigates through obstacle-dense environments in both simulation and real flight can be found at https://youtu.be/0awjK_5kGfw (accessed on 1 June 2023).

**Figure 16:**
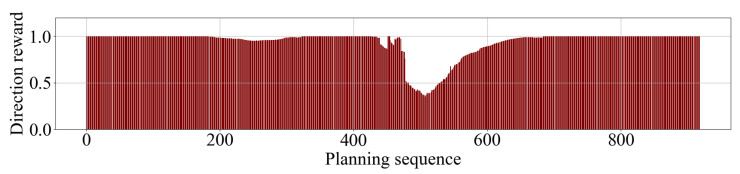
Path direction reward throughout a flight trial.

Results of calculated path’s direction rewards show that the path-planning algorithm works effectively on generated SFCs. When the robot is in front of a large free space, reward values are always 1 (maximum). They only decrease to a lower value when the vehicle is steering away from an obstacle on the route. Reward values saved from a trial are illustrated in Figure 16.

## 7. Conclusions

In this paper, we have presented a new method to generate high-quality safe flight corridors and a depth-based memoryless planner on top of it. We exploited the advantages of a learning-based object-detection model to produce large and less-overlapping corridors compared to a depth-based inflating method. Furthermore, an additional lightweight verification procedure has been adopted to discard false predictions, ensuring collision-free generated corridors. We then develop a memoryless local planner on top of that to verify the approach. The planner uses only depth frames, and it can plan the best direction paths to the goal. The whole system was implemented on an experimental quadrotor and can be run in real time with all sensing and computation onboard. Simulation and real flight-testing results show that our system can effectively employ the proposed SFC generator to navigate a quadrotor through a cluttered scenario safely.

One limitation of our method is that it can be conservative due to weak labeling and verification procedure. Hence, the generated SFCs may not represent free space exhaustively when the environment becomes extremely cluttered. This limitation can be improved by employing semi-supervised learning in the object-detection model. In future research, we will explore this technique and adapt it to more challenging scenarios.

## Figures and Tables

**Table 1:** SFC generation methods comparison with actual data.

Platform	SFC Generator	Overall IoU	Number of SFCs Generated per Frame	Average Planning Time	Average Direction Reward
Xavier NX	Exhaustive inflating	42.27%	16.53	78.16 ms	0.97
	Proposed **FSD**	**1.35%**	**5.88**	**26.73 ms**	**0.97**
i7 laptop	Exhaustive inflating	51.78%	56	4.05 ms	0.97
	Proposed **FSD**	**1.35%**	**5.88**	**0.44 ms**	**0.97**

**Table 2:** Success rate and average flight time of the planning system for three simulated scenarios.

	Success Rate	Average Flight Time
Easy	99.9%	23.8 s
Medium	99.8%	22.8 s
Hard	99.6%	23.9 s

## Data Availability

Available upon request.

## Abbreviations

The following abbreviations are used in this manuscript:

SFC	Safe flight corridor
FSD	Free space detection
IoU	Intersection over union
FoV	Field of view
